# (*E*)-5-[(1,5-Dimethyl-3-oxo-2-phenyl-2,3-dihydro-1*H*-pyrazol-4-yl)imino­meth­yl]-2-methoxy­phenyl 4-bromo­benzene­sulfonate

**DOI:** 10.1107/S1600536810017198

**Published:** 2010-05-15

**Authors:** Min-Jie Guo, Xin Chen, Jing-Xia Yao

**Affiliations:** a College of Sciences, Tianjin University of Science and Technology, Tianjin 300457, People’s Republic of China

## Abstract

In the title compound, C_25_H_22_BrN_3_O_5_S, the central benzene ring makes dihedral angles of 32.02 (14), 37.49 (18) and 80.52 (13)°, respectively, with the pyrazolone ring, the bromo­benzene ring and the terminal phenyl ring. This conformation features a short intramolecular C—H⋯O contact that generates an *S*(6) ring. In the crystal, inversion dimers linked by pairs of C—H⋯O=C hydrogen bonds occur.

## Related literature

For general background to Schiff bases, see: Santos *et al.* (2001 ▶). For related structures, see: Chen & Yu (2006 ▶); Zhang *et al.* (2006 ▶). For reference structural data, see: Allen *et al.* (1987 ▶).
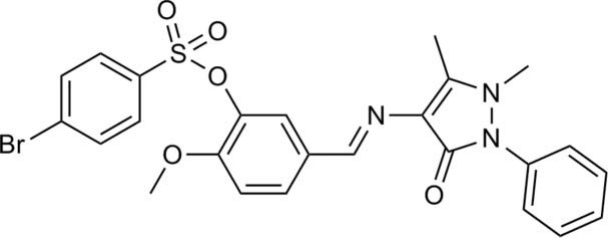


## Experimental

### 

#### Crystal data

C_25_H_22_BrN_3_O_5_S
*M* *_r_* = 556.43
Monoclinic, 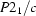

*a* = 11.102 (2) Å
*b* = 10.336 (2) Å
*c* = 22.160 (4) Å
β = 98.81 (3)°
*V* = 2512.9 (8) Å^3^
*Z* = 4
Mo *K*α radiation
μ = 1.76 mm^−1^
*T* = 294 K
0.24 × 0.20 × 0.12 mm

#### Data collection

Bruker SMART APEX CCD diffractometer
Absorption correction: multi-scan (*SADABS*; Sheldrick, 1996 ▶) *T*_min_ = 0.628, *T*_max_ = 0.810
12656 measured reflections
4431 independent reflections
2007 reflections with *I* > 2σ(*I*)
*R*_int_ = 0.080

#### Refinement

*R*[*F* ^2^ > 2σ(*F* ^2^)] = 0.057
*wR*(*F* ^2^) = 0.163
*S* = 1.00
4431 reflections
320 parameters
H-atom parameters constrained
Δρ_max_ = 0.98 e Å^−3^
Δρ_min_ = −0.82 e Å^−3^

### 

Data collection: *SMART* (Bruker, 1999 ▶); cell refinement: *SAINT* (Bruker, 1999 ▶); data reduction: *SAINT*; program(s) used to solve structure: *SHELXS97* (Sheldrick, 2008 ▶); program(s) used to refine structure: *SHELXL97* (Sheldrick, 2008 ▶); molecular graphics: *SHELXTL* (Sheldrick, 2008 ▶); software used to prepare material for publication: *SHELXTL*.

## Supplementary Material

Crystal structure: contains datablocks I, global. DOI: 10.1107/S1600536810017198/hb5442sup1.cif

Structure factors: contains datablocks I. DOI: 10.1107/S1600536810017198/hb5442Isup2.hkl

Additional supplementary materials:  crystallographic information; 3D view; checkCIF report

## Figures and Tables

**Table 1:** Hydrogen-bond geometry (Å, °)

*D*—H⋯*A*	*D*—H	H⋯*A*	*D*⋯*A*	*D*—H⋯*A*
C14—H14⋯O5	0.93	2.36	3.046 (7)	130
C9—H9⋯O5^i^	0.93	2.45	3.238 (6)	143

Symmetry code: (i) 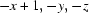.

**Fig. 1.**
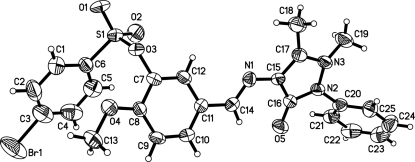
The structure of (I), with displacement ellipsoids for non-H atoms drawn at the 50% probability level.

**Fig. 2.**
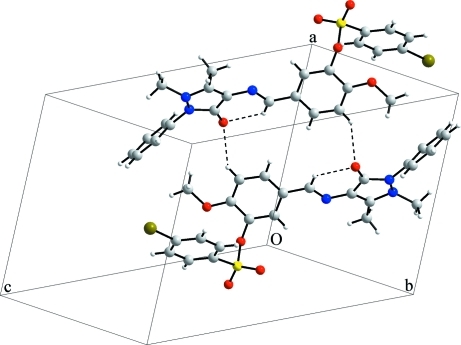
A packing diagram for (I), with hydrogen bonds drawn as dashed lines.

## supplementary crystallographic information

### Comment

There has been steady growth of interest in the synthesis, structure, and reactivity of Schiff bases due to their potentially biological activities such as protein and enzyme mimics (Santos *et al.*, 2001). Among the large number of compounds, 4-amino-1,5-dimethyl-2-phenylpyrazol-3-one forms a variety of Schiff bases with aldehydes, and the synthesis and crystal structures of some of them, such as (*E*)-5-(1,5-Dimethyl-3-oxo-2-phenyl-2,3-dihydro- 1*H*-pyrazol-4-yliminomethyl)-2-methoxyphenyl benzenesulfonate (Chen & Yu, 2006) and (*E*)-4-(2-(4-Chlorobenzyloxy)benzylideneamino) -2,3-dimethyl-1-phenyl-1,2-dihydropyrazol-5-one (Zhang *et al.*, 2006) have been reported.

Structural information is useful when investigating the coordination properties of Schiff bases functioning as ligands. We report here the synthesis and molecular structure of the title Schiff base compound, (I), (Fig. 1)

In the title molecule (Fig. 1), bond lengths and angles are within normal ranges (Allen *et al.*, 1987). The pyrazolone ring (C15—C17/N1—N3/O5) is almost planar, with an r.m.s. deviation for fitted atoms of 0.0331 Å. It makes a dihedral angle of 51.63 (17)° with the attached phenyl ring (C20—C25). The central benzene ring (C7—C12/C14/O3/O4) is nearly planar, with an r.m.s. deviation for fitted atoms of 0.0371 Å. This group makes dihedral angles of 32.02 (14)°, 37.49 (18)° and 80.52 (13)°, respectively, with the the pyrazolone ring (C15—C17/N1—N3/O5), the bromobenzene ring (C1—C6) and the terminal phenyl ring (C20—C25).

An intramolecular C14—H14···O5═C16 hydrogen bond is found in (I) (Table 1), which helps to stabilize the conformation of the molecule. Packing is stabilised by weak, non-classical intermolecular C9—H9···O5═C16 hydrogen bonds that form inversion related dimers (Table 1, Fig. 2).

### Experimental

An anhydrous ethanol solution (50 ml) of 5-formyl-2-methoxyphenyl 4-bromobenzenesulfonate (3.71 g, 10 mmol) was added to an anhydrous ethanol solution (50 ml) of 4-amino-1,5-dimethyl-2-phenylpyrazol-3-one (2.03 g, 10 mmol) and the mixture stirred at 350 K for 3 h under N_2_, giving a yellow precipitate. The product was isolated, recrystallized from acetonitrile, and then dried in a vacuum to give pure compound (I) in 83% yield. Yellow blocks of (I) were obtained by slow evaporation of an acetonitrile solution.

### Refinement

The H atoms were included in calculated positions and refined using a riding model approximation. Constrained C—H bond lengths and isotropic U parameters: 0.93 Å and U_iso_(H) = 1.2U_eq_(C) for Csp^2^—H; 0.96 Å and U_iso_(H) = 1.5U_eq_(C) for methyl C—H.

### Crystal data

**Table:**

C_25_H_22_BrN_3_O_5_S	*F*(000) = 1136
*M**_r_* = 556.43	*D*_x_ = 1.471 Mg m^−^^3^
Monoclinic, *P*2_1_/*c*	Mo *K*α radiation, λ = 0.71073 Å
Hall symbol: -P 2ybc	Cell parameters from 1629 reflections
*a* = 11.102 (2) Å	θ = 2.2–19.4°
*b* = 10.336 (2) Å	µ = 1.76 mm^−^^1^
*c* = 22.160 (4) Å	*T* = 294 K
β = 98.81 (3)°	Block, yellow
*V* = 2512.9 (8) Å^3^	0.24 × 0.20 × 0.12 mm
*Z* = 4	

### Data collection

**Table:**

Bruker SMART APEX CCD diffractometer	4431 independent reflections
Radiation source: fine-focus sealed tube	2007 reflections with *I* > 2σ(*I*)
graphite	*R*_int_ = 0.080
φ and ω scans	θ_max_ = 25.0°, θ_min_ = 1.9°
Absorption correction: multi-scan (*SADABS*; Sheldrick, 1996)	*h* = −8→13
*T*_min_ = 0.628, *T*_max_ = 0.810	*k* = −12→11
12656 measured reflections	*l* = −26→25

### Refinement

**Table:**

Refinement on *F*^2^	Secondary atom site location: difference Fourier map
Least-squares matrix: full	Hydrogen site location: inferred from neighbouring sites
*R*[*F*^2^ > 2σ(*F*^2^)] = 0.057	H-atom parameters constrained
*wR*(*F*^2^) = 0.163	*w* = 1/[σ^2^(*F*_o_^2^) + (0.0562*P*)^2^ + 2.7821*P*] where *P* = (*F*_o_^2^ + 2*F*_c_^2^)/3
*S* = 1.00	(Δ/σ)_max_ < 0.001
4431 reflections	Δρ_max_ = 0.98 e Å^−^^3^
320 parameters	Δρ_min_ = −0.82 e Å^−^^3^
0 restraints	Extinction correction: *SHELXL97* (Sheldrick, 2008), Fc^*^=kFc[1+0.001xFc^2^λ^3^/sin(2θ)]^-1/4^
Primary atom site location: structure-invariant direct methods	Extinction coefficient: 0.0027 (4)

### Special details

**Table:**

Geometry. All esds (except the esd in the dihedral angle between two l.s. planes) are estimated using the full covariance matrix. The cell esds are taken into account individually in the estimation of esds in distances, angles and torsion angles; correlations between esds in cell parameters are only used when they are defined by crystal symmetry. An approximate (isotropic) treatment of cell esds is used for estimating esds involving l.s. planes.
Refinement. Refinement of *F*^2^ against ALL reflections. The weighted *R*-factor wR and goodness of fit *S* are based on *F*^2^, conventional *R*-factors *R* are based on *F*, with *F* set to zero for negative *F*^2^. The threshold expression of *F*^2^ > σ(*F*^2^) is used only for calculating *R*-factors(gt) etc. and is not relevant to the choice of reflections for refinement. *R*-factors based on *F*^2^ are statistically about twice as large as those based on *F*, and *R*- factors based on ALL data will be even larger.

### Fractional atomic coordinates and isotropic or equivalent isotropic displacement parameters (Å^2^)

**Table:**

	*x*	*y*	*z*	*U*_iso_*/*U*_eq_	
Br1	−0.02396 (8)	−0.56145 (7)	0.12388 (5)	0.1174 (5)	
S1	−0.05698 (13)	0.05809 (14)	0.13190 (8)	0.0487 (4)	
N1	0.3100 (4)	0.3198 (4)	0.0051 (2)	0.0395 (11)	
N2	0.4162 (4)	0.4909 (4)	−0.1195 (2)	0.0452 (12)	
N3	0.3891 (4)	0.5927 (4)	−0.0811 (2)	0.0417 (12)	
O1	−0.1268 (4)	0.1008 (4)	0.1771 (2)	0.0718 (13)	
O2	−0.0793 (3)	0.1107 (4)	0.07154 (18)	0.0615 (12)	
O3	0.0813 (3)	0.0930 (3)	0.16212 (16)	0.0422 (9)	
O4	0.2066 (3)	−0.1252 (3)	0.19075 (17)	0.0508 (10)	
O5	0.4261 (3)	0.2660 (3)	−0.11424 (18)	0.0584 (11)	
C1	−0.0754 (5)	−0.1852 (6)	0.1783 (3)	0.0611 (18)	
H1	−0.0927	−0.1440	0.2133	0.073*	
C2	−0.0685 (6)	−0.3191 (7)	0.1770 (4)	0.070 (2)	
H2	−0.0824	−0.3682	0.2104	0.084*	
C3	−0.0402 (6)	−0.3780 (7)	0.1246 (4)	0.071 (2)	
C4	−0.0228 (6)	−0.3070 (7)	0.0741 (4)	0.069 (2)	
H4	−0.0055	−0.3483	0.0392	0.083*	
C5	−0.0314 (5)	−0.1730 (6)	0.0759 (3)	0.0566 (17)	
H5	−0.0200	−0.1240	0.0420	0.068*	
C6	−0.0571 (5)	−0.1124 (5)	0.1284 (3)	0.0449 (15)	
C7	0.1760 (4)	0.0594 (5)	0.1284 (2)	0.0356 (13)	
C8	0.2399 (4)	−0.0569 (5)	0.1435 (2)	0.0370 (13)	
C9	0.3316 (5)	−0.0897 (5)	0.1096 (3)	0.0421 (14)	
H9	0.3750	−0.1663	0.1178	0.050*	
C10	0.3579 (5)	−0.0081 (5)	0.0636 (3)	0.0449 (15)	
H10	0.4177	−0.0331	0.0408	0.054*	
C11	0.2986 (4)	0.1100 (5)	0.0501 (2)	0.0379 (13)	
C12	0.2050 (5)	0.1420 (5)	0.0842 (2)	0.0388 (14)	
H12	0.1628	0.2195	0.0767	0.047*	
C13	0.2697 (5)	−0.2459 (5)	0.2063 (3)	0.0636 (18)	
H13A	0.2586	−0.3022	0.1714	0.095*	
H13B	0.2374	−0.2863	0.2394	0.095*	
H13C	0.3551	−0.2292	0.2184	0.095*	
C14	0.3353 (5)	0.1982 (5)	0.0048 (3)	0.0429 (14)	
H14	0.3779	0.1660	−0.0250	0.052*	
C15	0.3472 (4)	0.4037 (5)	−0.0382 (2)	0.0361 (13)	
C16	0.3973 (5)	0.3721 (5)	−0.0931 (3)	0.0403 (14)	
C17	0.3399 (5)	0.5359 (5)	−0.0341 (2)	0.0406 (14)	
C18	0.2903 (5)	0.6155 (5)	0.0127 (3)	0.0573 (17)	
H18A	0.2546	0.5597	0.0397	0.086*	
H18B	0.2293	0.6736	−0.0072	0.086*	
H18C	0.3550	0.6645	0.0357	0.086*	
C19	0.3517 (6)	0.7179 (5)	−0.1087 (3)	0.0663 (19)	
H19A	0.2817	0.7063	−0.1394	0.099*	
H19B	0.4173	0.7539	−0.1269	0.099*	
H19C	0.3317	0.7757	−0.0778	0.099*	
C20	0.4861 (5)	0.5129 (5)	−0.1677 (3)	0.0415 (14)	
C21	0.4518 (5)	0.4494 (5)	−0.2229 (3)	0.0518 (16)	
H21	0.3847	0.3944	−0.2280	0.062*	
C22	0.5180 (6)	0.4683 (6)	−0.2703 (3)	0.0634 (19)	
H22	0.4958	0.4249	−0.3071	0.076*	
C23	0.6173 (7)	0.5514 (7)	−0.2633 (3)	0.074 (2)	
H23	0.6601	0.5658	−0.2957	0.089*	
C24	0.6519 (6)	0.6123 (6)	−0.2083 (3)	0.0669 (19)	
H24	0.7196	0.6664	−0.2034	0.080*	
C25	0.5879 (5)	0.5945 (5)	−0.1599 (3)	0.0543 (16)	
H25	0.6121	0.6362	−0.1229	0.065*	

### Atomic displacement parameters (Å^2^)

**Table:**

	*U*^11^	*U*^22^	*U*^33^	*U*^12^	*U*^13^	*U*^23^
Br1	0.1074 (7)	0.0431 (5)	0.1895 (12)	0.0027 (4)	−0.0163 (6)	−0.0076 (5)
S1	0.0404 (9)	0.0425 (8)	0.0666 (12)	0.0128 (7)	0.0188 (8)	0.0081 (8)
N1	0.038 (3)	0.042 (3)	0.040 (3)	−0.002 (2)	0.007 (2)	0.008 (2)
N2	0.054 (3)	0.035 (3)	0.053 (3)	0.006 (2)	0.027 (3)	0.001 (2)
N3	0.050 (3)	0.031 (3)	0.046 (3)	0.008 (2)	0.013 (2)	0.005 (2)
O1	0.063 (3)	0.065 (3)	0.099 (4)	0.025 (2)	0.048 (3)	0.002 (3)
O2	0.057 (3)	0.067 (3)	0.060 (3)	0.015 (2)	0.006 (2)	0.032 (2)
O3	0.041 (2)	0.037 (2)	0.052 (3)	0.0062 (16)	0.0158 (18)	−0.0046 (18)
O4	0.051 (2)	0.047 (2)	0.054 (3)	0.0093 (18)	0.010 (2)	0.016 (2)
O5	0.074 (3)	0.039 (2)	0.068 (3)	0.006 (2)	0.031 (2)	−0.004 (2)
C1	0.058 (4)	0.056 (4)	0.073 (5)	0.000 (3)	0.020 (4)	0.008 (4)
C2	0.062 (5)	0.053 (5)	0.093 (6)	−0.006 (3)	0.005 (4)	0.027 (4)
C3	0.049 (4)	0.049 (4)	0.107 (7)	0.000 (3)	−0.012 (4)	−0.004 (5)
C4	0.060 (5)	0.061 (5)	0.081 (6)	0.007 (3)	−0.007 (4)	−0.021 (4)
C5	0.046 (4)	0.064 (5)	0.055 (5)	0.001 (3)	−0.008 (3)	−0.001 (4)
C6	0.039 (3)	0.038 (3)	0.057 (4)	0.001 (3)	0.006 (3)	0.007 (3)
C7	0.039 (3)	0.029 (3)	0.039 (3)	0.006 (2)	0.005 (3)	−0.001 (3)
C8	0.036 (3)	0.031 (3)	0.043 (4)	−0.001 (2)	0.003 (3)	0.005 (3)
C9	0.039 (3)	0.029 (3)	0.057 (4)	0.009 (2)	0.003 (3)	−0.004 (3)
C10	0.041 (3)	0.042 (3)	0.056 (4)	0.007 (3)	0.019 (3)	−0.001 (3)
C11	0.034 (3)	0.037 (3)	0.044 (4)	−0.001 (2)	0.009 (3)	0.001 (3)
C12	0.040 (3)	0.028 (3)	0.047 (4)	0.003 (2)	0.004 (3)	0.000 (3)
C13	0.069 (4)	0.045 (4)	0.076 (5)	0.014 (3)	0.009 (3)	0.020 (4)
C14	0.040 (4)	0.044 (3)	0.048 (4)	0.003 (3)	0.017 (3)	0.002 (3)
C15	0.030 (3)	0.035 (3)	0.044 (4)	0.005 (2)	0.007 (3)	−0.001 (3)
C16	0.039 (3)	0.034 (3)	0.049 (4)	0.002 (3)	0.010 (3)	0.003 (3)
C17	0.041 (3)	0.040 (3)	0.040 (4)	0.004 (3)	0.003 (3)	0.001 (3)
C18	0.066 (4)	0.048 (4)	0.061 (4)	0.008 (3)	0.016 (3)	−0.001 (3)
C19	0.085 (5)	0.041 (4)	0.078 (5)	0.018 (3)	0.029 (4)	0.016 (3)
C20	0.043 (4)	0.042 (3)	0.040 (4)	0.008 (3)	0.009 (3)	0.007 (3)
C21	0.052 (4)	0.056 (4)	0.048 (4)	0.011 (3)	0.009 (3)	0.004 (3)
C22	0.074 (5)	0.077 (5)	0.040 (4)	0.028 (4)	0.010 (4)	0.004 (4)
C23	0.082 (5)	0.089 (5)	0.061 (5)	0.024 (5)	0.040 (4)	0.031 (5)
C24	0.060 (4)	0.074 (5)	0.071 (5)	−0.003 (3)	0.023 (4)	0.012 (4)
C25	0.057 (4)	0.054 (4)	0.054 (4)	−0.003 (3)	0.016 (3)	0.004 (3)

### Geometric parameters (Å, °)

**Table:**

Br1—C3	1.905 (7)	C9—H9	0.9300
S1—O1	1.427 (4)	C10—C11	1.398 (7)
S1—O2	1.430 (4)	C10—H10	0.9300
S1—O3	1.619 (4)	C11—C12	1.415 (7)
S1—C6	1.764 (6)	C11—C14	1.461 (7)
N1—C14	1.287 (6)	C12—H12	0.9300
N1—C15	1.403 (6)	C13—H13A	0.9600
N2—C16	1.390 (6)	C13—H13B	0.9600
N2—N3	1.414 (6)	C13—H13C	0.9600
N2—C20	1.433 (7)	C14—H14	0.9300
N3—C17	1.378 (6)	C15—C17	1.373 (7)
N3—C19	1.464 (6)	C15—C16	1.449 (7)
O3—C7	1.423 (6)	C17—C18	1.495 (7)
O4—C8	1.359 (6)	C18—H18A	0.9600
O4—C13	1.446 (6)	C18—H18B	0.9600
O5—C16	1.253 (6)	C18—H18C	0.9600
C1—C6	1.378 (8)	C19—H19A	0.9600
C1—C2	1.386 (8)	C19—H19B	0.9600
C1—H1	0.9300	C19—H19C	0.9600
C2—C3	1.389 (9)	C20—C21	1.388 (7)
C2—H2	0.9300	C20—C25	1.399 (7)
C3—C4	1.375 (9)	C21—C22	1.386 (8)
C4—C5	1.389 (8)	C21—H21	0.9300
C4—H4	0.9300	C22—C23	1.387 (9)
C5—C6	1.388 (8)	C22—H22	0.9300
C5—H5	0.9300	C23—C24	1.374 (9)
C7—C12	1.374 (7)	C23—H23	0.9300
C7—C8	1.410 (6)	C24—C25	1.386 (8)
C8—C9	1.397 (7)	C24—H24	0.9300
C9—C10	1.388 (7)	C25—H25	0.9300
			
O1—S1—O2	120.2 (3)	C11—C12—H12	119.8
O1—S1—O3	102.9 (2)	O4—C13—H13A	109.5
O2—S1—O3	108.7 (2)	O4—C13—H13B	109.5
O1—S1—C6	110.1 (3)	H13A—C13—H13B	109.5
O2—S1—C6	109.9 (3)	O4—C13—H13C	109.5
O3—S1—C6	103.6 (2)	H13A—C13—H13C	109.5
C14—N1—C15	120.8 (5)	H13B—C13—H13C	109.5
C16—N2—N3	110.2 (4)	N1—C14—C11	121.4 (5)
C16—N2—C20	126.0 (4)	N1—C14—H14	119.3
N3—N2—C20	121.4 (4)	C11—C14—H14	119.3
C17—N3—N2	106.4 (4)	C17—C15—N1	122.9 (5)
C17—N3—C19	124.9 (4)	C17—C15—C16	108.3 (5)
N2—N3—C19	118.6 (4)	N1—C15—C16	128.7 (4)
C7—O3—S1	117.3 (3)	O5—C16—N2	123.5 (5)
C8—O4—C13	116.9 (4)	O5—C16—C15	131.6 (5)
C6—C1—C2	120.9 (7)	N2—C16—C15	104.8 (4)
C6—C1—H1	119.6	C15—C17—N3	109.9 (5)
C2—C1—H1	119.6	C15—C17—C18	128.7 (5)
C1—C2—C3	118.4 (7)	N3—C17—C18	121.4 (5)
C1—C2—H2	120.8	C17—C18—H18A	109.5
C3—C2—H2	120.8	C17—C18—H18B	109.5
C4—C3—C2	121.5 (7)	H18A—C18—H18B	109.5
C4—C3—Br1	119.9 (6)	C17—C18—H18C	109.5
C2—C3—Br1	118.5 (6)	H18A—C18—H18C	109.5
C3—C4—C5	119.3 (7)	H18B—C18—H18C	109.5
C3—C4—H4	120.3	N3—C19—H19A	109.5
C5—C4—H4	120.3	N3—C19—H19B	109.5
C6—C5—C4	119.8 (6)	H19A—C19—H19B	109.5
C6—C5—H5	120.1	N3—C19—H19C	109.5
C4—C5—H5	120.1	H19A—C19—H19C	109.5
C1—C6—C5	120.0 (6)	H19B—C19—H19C	109.5
C1—C6—S1	120.6 (5)	C21—C20—C25	120.1 (5)
C5—C6—S1	119.3 (5)	C21—C20—N2	118.4 (5)
C12—C7—C8	122.4 (5)	C25—C20—N2	121.5 (5)
C12—C7—O3	119.6 (4)	C22—C21—C20	119.7 (6)
C8—C7—O3	117.9 (4)	C22—C21—H21	120.2
O4—C8—C9	126.5 (5)	C20—C21—H21	120.2
O4—C8—C7	116.1 (5)	C21—C22—C23	120.5 (6)
C9—C8—C7	117.4 (5)	C21—C22—H22	119.7
C10—C9—C8	120.0 (5)	C23—C22—H22	119.7
C10—C9—H9	120.0	C24—C23—C22	119.4 (6)
C8—C9—H9	120.0	C24—C23—H23	120.3
C9—C10—C11	122.9 (5)	C22—C23—H23	120.3
C9—C10—H10	118.6	C23—C24—C25	121.3 (6)
C11—C10—H10	118.6	C23—C24—H24	119.4
C10—C11—C12	116.8 (5)	C25—C24—H24	119.4
C10—C11—C14	121.4 (5)	C24—C25—C20	119.0 (6)
C12—C11—C14	121.8 (5)	C24—C25—H25	120.5
C7—C12—C11	120.4 (5)	C20—C25—H25	120.5
C7—C12—H12	119.8		
			
C16—N2—N3—C17	−6.8 (5)	O3—C7—C12—C11	−179.8 (4)
C20—N2—N3—C17	−170.1 (5)	C10—C11—C12—C7	0.3 (7)
C16—N2—N3—C19	−154.0 (5)	C14—C11—C12—C7	−177.2 (5)
C20—N2—N3—C19	42.7 (7)	C15—N1—C14—C11	179.2 (4)
O1—S1—O3—C7	178.7 (3)	C10—C11—C14—N1	−158.1 (5)
O2—S1—O3—C7	−52.8 (4)	C12—C11—C14—N1	19.3 (8)
C6—S1—O3—C7	64.0 (4)	C14—N1—C15—C17	−169.3 (5)
C6—C1—C2—C3	1.2 (10)	C14—N1—C15—C16	11.0 (8)
C1—C2—C3—C4	−1.9 (10)	N3—N2—C16—O5	−172.4 (5)
C1—C2—C3—Br1	177.4 (5)	C20—N2—C16—O5	−10.1 (9)
C2—C3—C4—C5	1.2 (10)	N3—N2—C16—C15	4.5 (5)
Br1—C3—C4—C5	−178.1 (4)	C20—N2—C16—C15	166.9 (5)
C3—C4—C5—C6	0.2 (9)	C17—C15—C16—O5	175.9 (6)
C2—C1—C6—C5	0.1 (9)	N1—C15—C16—O5	−4.3 (9)
C2—C1—C6—S1	−176.2 (5)	C17—C15—C16—N2	−0.6 (6)
C4—C5—C6—C1	−0.8 (8)	N1—C15—C16—N2	179.1 (5)
C4—C5—C6—S1	175.5 (4)	N1—C15—C17—N3	176.6 (4)
O1—S1—C6—C1	−25.1 (6)	C16—C15—C17—N3	−3.6 (6)
O2—S1—C6—C1	−159.7 (4)	N1—C15—C17—C18	−2.3 (9)
O3—S1—C6—C1	84.3 (5)	C16—C15—C17—C18	177.5 (5)
O1—S1—C6—C5	158.6 (4)	N2—N3—C17—C15	6.3 (6)
O2—S1—C6—C5	24.0 (5)	C19—N3—C17—C15	150.8 (5)
O3—S1—C6—C5	−91.9 (5)	N2—N3—C17—C18	−174.7 (5)
S1—O3—C7—C12	84.6 (5)	C19—N3—C17—C18	−30.1 (8)
S1—O3—C7—C8	−97.6 (5)	C16—N2—C20—C21	59.0 (7)
C13—O4—C8—C9	−2.1 (7)	N3—N2—C20—C21	−140.4 (5)
C13—O4—C8—C7	179.0 (4)	C16—N2—C20—C25	−120.0 (6)
C12—C7—C8—O4	175.9 (5)	N3—N2—C20—C25	40.6 (7)
O3—C7—C8—O4	−1.8 (7)	C25—C20—C21—C22	−0.7 (8)
C12—C7—C8—C9	−3.1 (7)	N2—C20—C21—C22	−179.7 (5)
O3—C7—C8—C9	179.2 (4)	C20—C21—C22—C23	−0.8 (9)
O4—C8—C9—C10	−178.0 (5)	C21—C22—C23—C24	1.9 (9)
C7—C8—C9—C10	0.9 (7)	C22—C23—C24—C25	−1.5 (10)
C8—C9—C10—C11	1.9 (8)	C23—C24—C25—C20	0.0 (9)
C9—C10—C11—C12	−2.5 (8)	C21—C20—C25—C24	1.1 (8)
C9—C10—C11—C14	175.0 (5)	N2—C20—C25—C24	−179.9 (5)
C8—C7—C12—C11	2.5 (8)		

### Hydrogen-bond geometry (Å, °)

**Table:**

*D*—H···*A*	*D*—H	H···*A*	*D*···*A*	*D*—H···*A*
C14—H14···O5	0.93	2.36	3.046 (7)	130
C9—H9···O5^i^	0.93	2.45	3.238 (6)	143

Symmetry codes: (i) −*x*+1, −*y*, −*z*.
